# Medium-chain chlorinated paraffins (MCCPs) induce renal cell aging and ferroptosis

**DOI:** 10.18632/aging.205756

**Published:** 2024-04-19

**Authors:** Dong Zhang, Zongying Li, Yuan Gao, Hailing Sun

**Affiliations:** 1 The First Department of Nephrology, Cangzhou Central Hospital, Cangzhou, Hebei, China; 2 Department of Hematology, Cangzhou Central Hospital, Cangzhou, Hebei, China

**Keywords:** MCCPs, premature aging, kidney, ferroptosis, inflammatory response

## Abstract

Purpose: Medium-chained chlorinated paraffins (MCCPs) are a class of chlorinated derivatives of straight-chain n-alkanes with complex compositions, which are widely used in industry. The chlorinated paraffins (CPs) are divided into short chain chlorinated paraffins (SCCPs), medium chain chlorinated paraffins (MCCPs) and long chain chlorinated paraffins (LCCPs). SCCPs have been banned due to their severe bioaccumulation and biotoxicity. Therefore, MCCPs are used as a substitute for SCCPs. However, the toxicological data of MCCPs are still very limited. For this, we systematically investigated the toxicological impact of MCCPs on a renal cell model in the current study. Our work provides basic research data for analyzing the toxicological effects of MCCPs, suggesting that MCCPs should be restricted in their usage.

Method: A series of biochemical experiments was performed, including Western blot, indirect immunofluorescence assay, and ELISA was performed to analyze the toxicological effects of MCCPs.

Results: Two renal cell lines were used as a model for assessing the toxicological effects of MCCPs. Cell proliferation assays showed that MCCPs could inhibit the proliferation of kidney cells in a dose-dependent manner. Further studies showed that MCCPs induced ferroptosis in kidney cells by evaluating a series of ferroptosis marker molecules. Additionally, MCCPs induced inflammatory response and premature senescence in HEK293 and NRK-52E cells. Molecular mechanism experiments showed that ferroptosis induced by MCCPs emerged as a significant contributor to premature aging of kidney cells.

Conclusion: The current study provides basic research data to analyze the toxicological effects of MCCPs and their toxicity mechanisms. It also provides a theoretical basis for the assessment of the potential ecological risk of MCCPs, as well as basic experimental data for the rational and standardized use of MCCPs.

## INTRODUCTION

Chlorinated paraffins (CPs) are a class of complex chlorinated derivatives of straight-chain n-alkanes with a wide range of industrial applications. CPs are bioaccumulative, biotoxic, and long-range migratory [1]. However, because CPs have the advantages of chemical stability, flame retardancy and low volatility, hundreds of products containing CPs have been developed and applied on a large scale so far, such as lubricants, plastic plasticizers, flame retardants, coating additives and so on [2]. According to the carbon chain length, CPs can be classified into short-chained chlorinated paraffins (SCCPs, C10-13), medium-chained chlorinated paraffins (MCCPs, C14-17) and long-chained chlorinated paraffins (LCCPs, C18-30) [3]. Due to the wide industrial application of CPs, CPs can be commonly detected in the environment and in living organisms. CPs exhibit the potential health hazards to animals, plants, and humans [4]. In the recent years, domestic and foreign scholars have paid more attention to CPs, and the assessment of ecological and human health risks of CPs has been widely carried out. SCCPs are characterized by long-range transport capacity, environmental persistence and bioaccumulation [5]. A series of progresses have been made in the study of the toxicology of SCCPs in animals. For example, the half-life and tissue distribution characteristics of SCCPs were revealed in a previous study using rats as a model animal [6], and SCCPs have been detected in many environmental media, such as soil and water [7]. There are many ways for humans to ingest SCCPs, including diet and inhalation of air and dust, etc. SCCPs can be ingested through a number of routes, such as consumption of fish enriched with SCCPs, which may pose potential human health risks [8]. Furthermore, SCCPs can be detected in human breast milk [9]. SCCPs were listed in the Stockholm Convention on Persistent Organic Pollutants (POPs) in 2017 [10].

MCCPs have been introduced into the market as a preferred alternative to SCCPs and are widely used. However, the biotoxicity of MCCPs has been gradually recognized and a lot of research has been carried out. With the widespread ban of SCCPs, the use of MCCPs has gradually increased, some studies have been conducted by researchers regarding the toxicology of MCCPs on animal and cellular models. Researchers have found that MCCPs can also cause a wide range of biological toxicity, including hepatotoxicity, developmental toxicity, carcinogenicity, endocrine and metabolic disruption. For example, Chen et al. found that MCCPs inhibited the proliferation of somatic cells [11]. Ren et al. reported that MCCPs caused toxic effects on liver cells [12]. However, up to now, the study of the toxic effects of MCCPs is very limited, so it is of great significance to carry out in-depth research on the toxic effects and mechanisms of MCCPs. More recently, an important study from Zhao et al. found that serum SCCP and MCCP concentrations are positively correlated with eGFR and glomerular hyperfiltration (GH) in males [13]. Geng et al. found that SCCP induced hepatotoxicity in male rat [14].

The kidney is an important target organ for toxicological studies. Therefore, in the current work two kidney cells were used to study the toxicological effects of MCCPs. Additionally, *in vivo* models were also established for studying the toxicological effects of MCCPs. We evaluated in depth the toxicological effects of MCCPs on kidney cells in this work. The present work provides basic research data for analyzing the toxicological effects of MCCPs and their toxicity mechanisms, as well as a theoretical basis for evaluating the potential ecological risks of MCCPs, and provides basic experimental data for rational and standardized use of MCCPs.

## METHODS

### Antibodies and reagents

Protease phosphatase inhibitor mixture, BCA protein concentration assay kit, DCFH-DA reactive oxygen species assay kit and RIPA lysate were purchased from Beyotime Biotechnology Company (Shanghai, China). DMEM cell culture, FBS (fetal bovine serum), BSA (bovine serum albumin) and ECL kit were purchased from Themo Fisher Scientific company (Shanghai, China). p21 polyclonal antibody (Cat No. PA1-4185) was purchased from Themo Fisher Scientific company (Shanghai, China). p21 (Cat No. 28248-1-AP), p16 (Cat No. 10883-1-AP) and GPX4 (Cat No. 67763-1-Ig) were from Proteintech company (Wuhan, China). Anti-p53 (Cat No. 60283-2-Ig), Mouse IL-6 ELISA Kit (Cat No. KE10007), TNF Alpha Monoclonal antibody (Cat No. 60291-1-Ig), Rat IL-1 beta ELISA Kit (Cat No: KE20021) and HMGB1 Polyclonal Antibody (Cat No. 10829-1-AP) were from Proteintech company (Wuhan, China). Ferrostatin-1 (Fer-1) and DFO (Deferoxamine) were purchased from MCE (Shanghai, China). Anti-Ferritin Heavy Chain (FTH) antibody (sc-376594) and anti-NCOA4 (sc-373739) were from Santa Cruz Biotechnology company (USA). x-CT polyclonal antibody (ab307601) was from Abcam (Shanghai, China). Malondialdehyde (MDA) assay kit and GSH assay kit were purchased from Nanjing Jiancheng Research Institute (China). The TMRE mitochondrial membrane potential assay kit was purchased from Beyotime company (Shanghai, China). The Fe^2+^ detection probe (FerroOrange) was purchased from Dojindo Chemical company (Kumamoto, Japan). Ferrous ion content assay kit (Cat No. BC5415, purchased from Solarbio Biotechnology, Beijing, China) was used to detect the levels of ferrous ions in the tissue.

### NRK 52 cell and HEK293 cell culture

The human HEK293 and Rat NRK cells were from the National Collection of Authenticated Cell Cultures (Shanghai, China). HEK293 and Rat NRK cells were cultured in Dulbecco’s Modified Eagle’s medium (DMEM, Gibco, USA) with 10% FBS.

### Cell senescence analysis by Sa-β-galactosidase staining

Kidney cells (NRK-52 cell and human HEK293) were inoculated into cell culture plates. When kidney cells grew to 75% confluence, different concentrations of MCCP were added (the selection of the MCCP concentration currently used is based on previous reports in the literature [12]. After incubation for 24 h, Sa-β-gal staining solution was added. After incubation for 3 h, cell samples were examined using a laser confocal microscope (FV3000).

### Cell proliferation analysis

Kidney cells (1 × 10^4^) were inoculated into 96-well culture plates. Cells were cultured for 24 h. Cells were then stimulated with MCCPs for 24 h. CCK-8 (10 μl per well) solution was added to each well and the cells were placed in a CO_2_ incubator to continue the incubation for 4 h. The absorbance of the cell samples was detected using a microplate reader (wavelength 450 nm).

### Western blot analysis

After treatment with MCCPs, cells were washed three times using PBS buffer and then cells were lysed. Cell lysates were collected by using a cell scraper and cell lysates were transferred to 1.5 ml EP tubes and centrifuged at 4°C for 10 min (12000 rpm). The supernatant was collected and the BCA kit was used to assay the concentration of proteins according to the kit instructions. Samples were electrophoresed and proteins were transferred to PVDF membranes. After washing, the PVDF membrane was placed in 5% BSA solution for 60 min. Primary antibody was added and incubated for 12 h at 4°C. The primary antibody was added to the PVDF membranes and incubated for 12 h at 4°C. After washing, secondary antibody was added and then the PVDF membrane was exposed using ECL luminescent solution.

### Intracellular reactive oxygen species (ROS) assay

Kidney cells were inoculated with 6-well cell culture plates. Cells were cultured for 24 hours. Rat NRK cells and HEK293 were challenged with MCCPs for indicated time points. After washing, the cells were incubated with ROS fluorescent probe (DCFH-DA, 10 μM/L). After incubation for 0.5 h, the cells were examined using a laser confocal microscope (FV3000).

### Indirect immunofluorescence

NRK-52 cells and HEK293 cells were inoculated in cell culture plates (1 × 10^4^ cells per well). Cells were incubated for 12 h, after which, PBS buffer was used to wash the cells twice (3 min each time). Cells were fixed using 4% paraformaldehyde for 0.5 h. Cells were then blocked using 1% BSA. Cells were washed using PBS buffer for 3 times (5 min each). Corresponding primary antibodies were added and incubated at 4°C for 16 h. After three washes, fluorescently labeled secondary antibodies were added and incubated at room temperature away from light for 60 min. After washing with PBS, DAPI staining solution was added dropwise to stain the nuclei of the cells. The cells were then incubated at room temperature away from light for 15 min. After three washes, the cells were visualized using a laser confocal microscope (FV3000).

### MitoSOX™ staining

Rat NRK-52 cells and HEK293 cells (1.5 × 10^5^/well) were inoculated in 6-well plates. When the cells grew to 80% to 85% fusion, the cells were stimulated with MCCPs and placed in an incubator at 37°C in 5% CO_2_ for 6 h. After washing, MitoSOX™ probe was added (final concentration of 5 μM) sequentially to the 6-well plates. Cells were placed in an incubator (37°C, 5% CO_2_) for 20 min. After washing, cell samples were detected (excitation wavelength 510 nm, emission wavelength 580 nm).

### Detection of iron ion level

Kidney cells were inoculated in 12-well cell culture plates (containing cell slides). When the adherent cells were fused to about 70%, MCCP was used to stimulate the cells for the indicated time points. The cell culture medium was discarded and PBS was used to rinse the cells. Ferro-Orange working solution at a concentration of 1 μmol/l was added and incubated at 37°C in a 5% CO_2_ incubator. Cell samples were examined under a laser confocal microscope.

### Intracellular MDA/GSH assay

Kidney cells (NRK-52 cells and HEK293 cells) were inoculated in cell culture plates. When the cells were fused to 80% confluence. The NRK-52 cells and HEK293 cells were treated with MCCP for the indicated time points. Cells were collected into 1.5 mL centrifuge tubes. Cells were subjected to fragmentation using an ultrasonic fragmenter. Cell suspension samples were centrifuged at 12000 r/min for 15 min. Supernatants were extracted and protein concentrations were determined. The levels of MDA/GSH were subsequently determined using the MDA and GSH assay kit according to the manufacturer’s instructions.

### Analysis of mitochondrial membrane potential

Rat NRK-52 cells and human HEK293 cells (1.5 × 10^5^/well) were inoculated in 12-well plates. Rat NRK-52 cells and human HEK293 cells were challenged with MCCPs for different time points. After incubation, the cells were washed three times using PBS, samples of NRK-52 cells and HEK293 cells were examined using a laser confocal microscope (Olympus FV-3000).

### Cell cycle assay

Cells were processed after MCCPs. Cells were washed twice with pre-cooled PBS. Cells were collected by centrifugation (1,500 rpm, 5 min). 100 μL of PBS solution was added to cell precipitate to resuspend the cells. Cells were then fixed using 70% ethanol. After cell fixation, cells were centrifuged in a centrifuge at 1,500 rpm for 5 min. Cells were washed again using pre-cooled PBS. 100 μL of RNase (50 μg/mL) was added to resuspend the cells, and the cells were placed at 37°C for 30 min. 400 ul of PI (50 μg/mL) was added, and the cells were placed at 4°C for 30 min.

### Animal experiments

All animal experiments were approved by the Animal Care and Use Committee of Cangzhou Central Hospital (No. 2021-279-01). C57 mice (8 weeks old) were housed in a controlled environment (22 ± 3°C, 12 h light/dark cycle). Mice were acclimatized for one week prior to the experiment. Mice were randomly assigned to groups (10 mice per group). Mice were treated with different doses (0, 0.01, 1 and 100 mg/kg/d) of MCCPs for 4 weeks [13]. In the MCCP-treated group, MCCPs were dissolved in corn oil. The mice received MCCPs by gavage. Mice in the control group received only corn oil treatment. Mouse tissues were collected for histopathological examination at the end of the experiment, and the remaining tissues were frozen with liquid nitrogen and stored at −80°C for use in the corresponding experiments.

### Immunohistochemical experiments

Tissue sections were permeabilized with 0.2% Triton-X100 (Triton-X100 dissolved in 0.01 mol/L PBS) for 10 min. 10% goat serum was added dropwise to seal the sections and incubated at room temperature for 20 min. Primary antibodies were added (dilution ratio 1:500) at 4°C overnight, the negative control samples were incubated with PBS. Sections were washed three times (5 min each), fluorescently labeled secondary antibodies were added dropwise and incubated at room temperature for 20 min. Sections were washed three times with PBS (5 min each). The samples were detected with a microscope.

### Statistical analysis

The experimental data were expressed as Mean ± Standard Deviation (Mean ± SD) and the data were statistically processed using GraphPad Prism 8 statistical software. The Shapiro—Wilk test was applied to test for a normal distribution, comparisons of differences between two groups were analyzed by *t*-test, and comparisons between multiple groups were analyzed by one-way ANOVA (One-way ANOVA). *P* < 0.05 indicates that the difference is statistically significant (^*^P < 0.05; ^**^P < 0.01; ^***^P < 0.001).

### Data availability

The data supporting this study’s findings are available on request from the corresponding author.

## RESULTS

### Effect of MCCPs on the proliferation of NRK-52E cells and HEK293 cells by CCK8 assay

**Figure 1:**
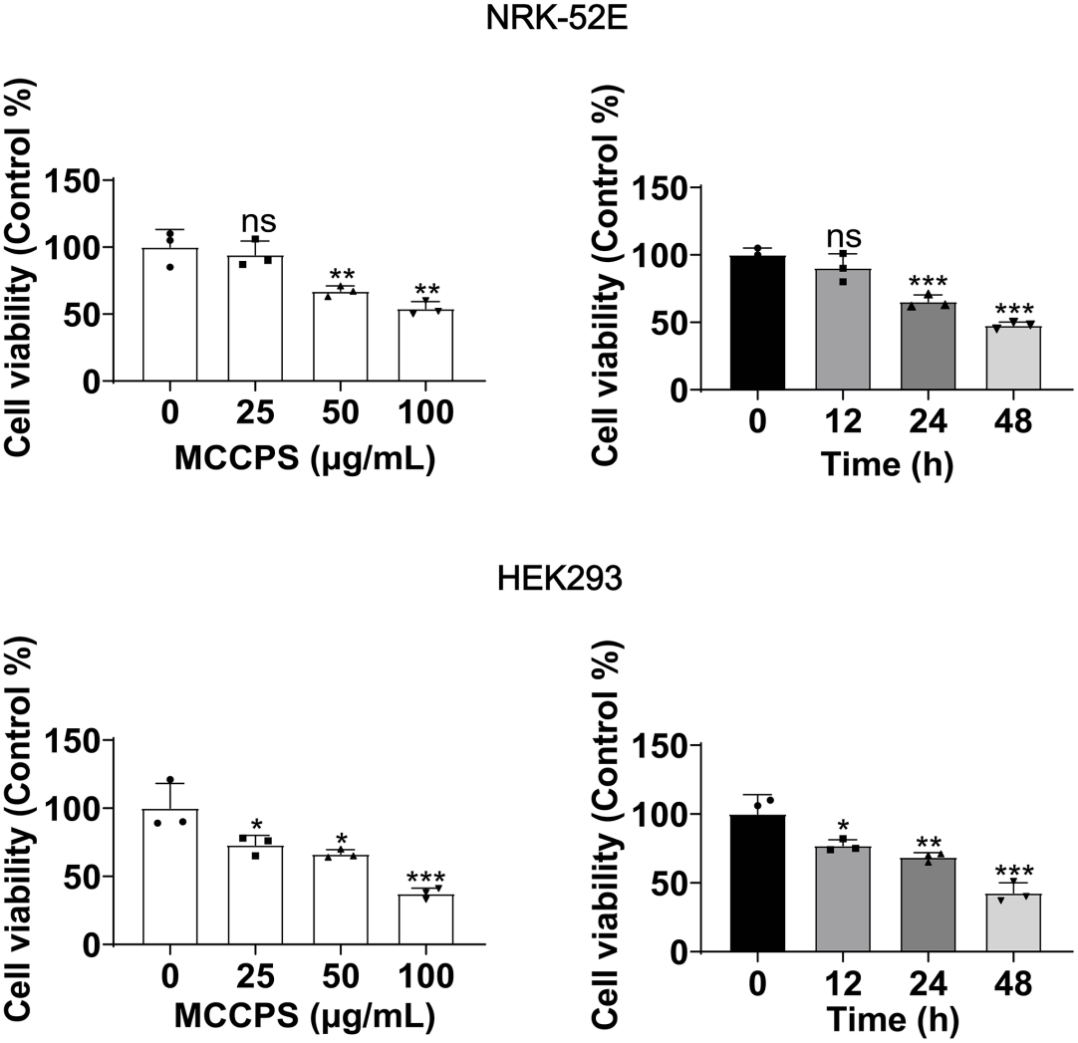
**Effect of MCCPs on renal cell viability.** The effect of MCCPs on the proliferative capacity of kidney cells was detected by CCK8. *p* < 0.05 indicates significant difference.

Here, we evaluated the effects of MCCPs on the proliferation of NRK-52E and HEK293 cells by treating cells with 0 μg/L, 25 μg/L, 50 μg/L and 100 μg/L of MCCPs for 24 h (The selection of the concentration of MCCPs was based on a previous literature reported [12]), the results showed that the renal cell viability decreased dose-dependently with the increase of the concentration of MCCPs, and that 50 μg/mL MCCPs obviously inhibited the proliferative viability of NRK-52E cell and HEK293. Next, NRK-52E and HEK293 cells were selected to be treated with 50 μg/mL MCCPs for 0 h, 12 h, 24 h and 48 h, it was found that the proliferative activity of NRK-52E and HEK293 cells was gradually decreased with the prolongation of the stimulation time of MCCPs (Figure 1).

### Effect of MCCPs on inflammation of Rat NRK-52E cell and HEK293

**Figure 2:**
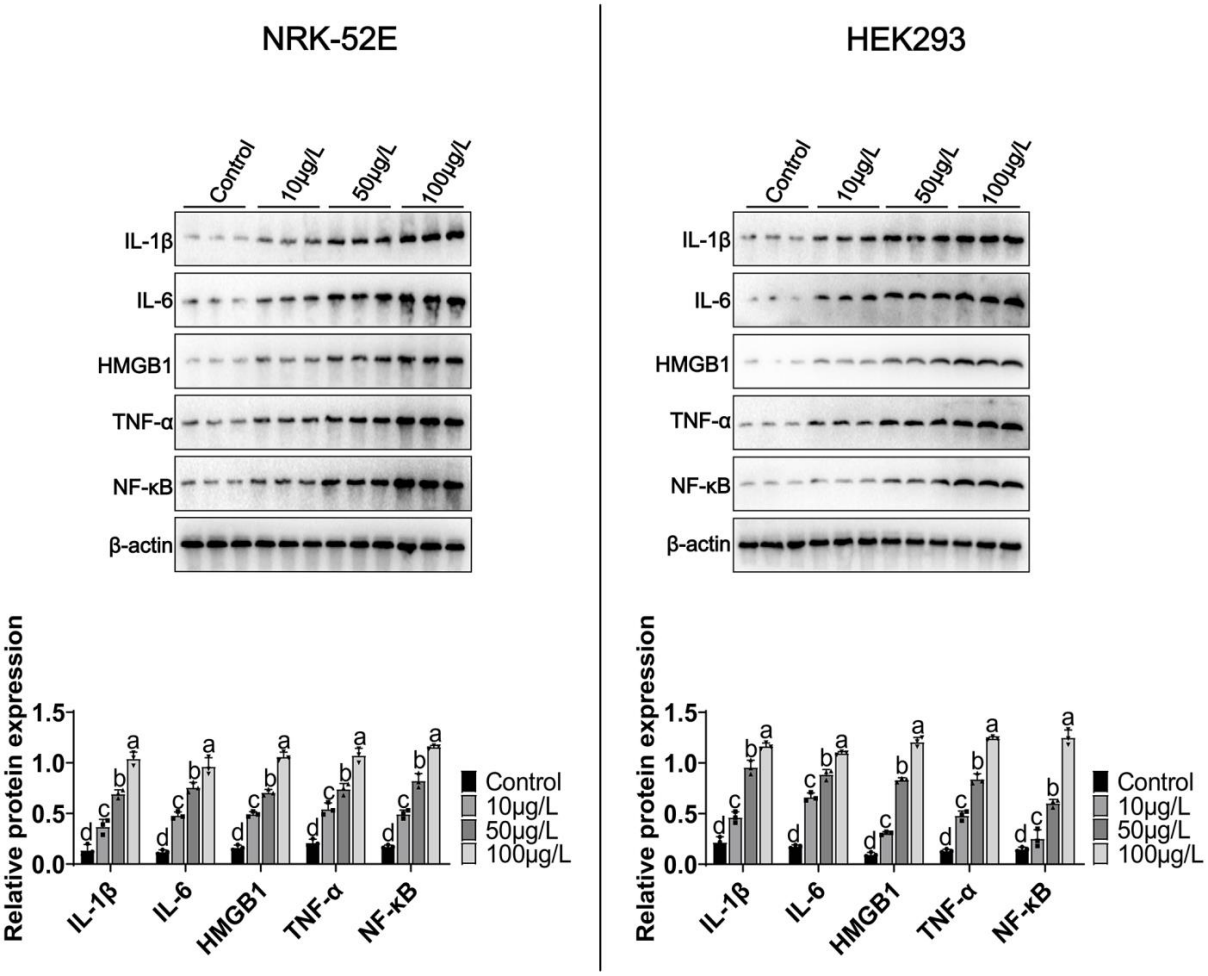
**Effects of MCCPs on inflammation in NRK-52E and HEK293 cells.** Western blot analysis of the effects of MCCPs on the expression of inflammatory factors. *p* < 0.05 indicates significant differences.

We treated NRK-52E/HEK293 with different concentrations of MCCPs (0 μg/L-100 μg/L) for 24 h. The experimental data demonstrated that MCCPs were able to significantly up-regulate the expression of inflammatory molecules, including IL-β, HMGB1, IL-6 and TNF-α expression (Figure 2). In addition, the expression of the inflammatory signaling molecule NF-κB was also significantly elevated compared to control group. These data suggest that MCCPs can induce inflammatory responses in NRK-52E and HEK293 cells (Figure 2).

### MCCPs induced senescence in Rat NRK-52E cell and HEK293 cells

**Figure 3:**
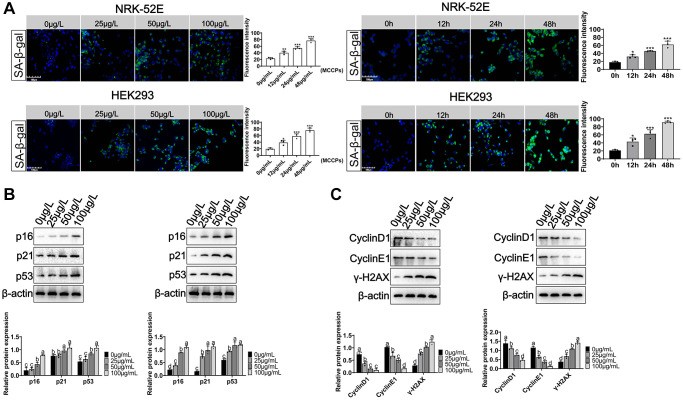
**Effects of MCCPs on renal cell senescence.** (**A**) Sa-β-gal staining of NRK-52E and HEK293 cells after MCCPs treatment. (**B**) Effects of MCCPs on p16/p21/p53 expression in NRK-52E and HEK293 cell model. (**C**) Effect of MCCPs on the expression of CyclinD1, CyclinE1 and γ-H2AX. *p* < 0.05 indicates significant difference.

Here, we evaluated the effect of MCCPs on renal cell senescence. NRK-52E and HEK293 cells were treated with 0 μg/L, 25 μg/L, 50 μg/L and 100 μg/L MCCPs for 24 h. Sa-β-gal staining showed that MCCPs caused the senescence of kidney cells. Additionally, NRK-52E and HEK293 cells were treated with 50 μg/L MCCPs for 0 h, 12 h, 24 h and 48 h. SPiDER-βGal staining showed that MCCPs were able to induce cellular senescence in NRK-52E cell and HEK293 cells in a time-dependent manner (Figure 3A). The effects of MCCPs on senescence-associated molecular markers were evaluated, including p21, p53, p16, γ-H2AX, CyclinD1 and CyclinE1. The experimental data showed that MCCPs induced an increase in the expression of p16/p21/p53. MCCPs (50 μg/L) was used to treat NRK-52E and HEK293 cells for 0 h, 12 h, 24 h and 48 h. The experimental data showed that MCCPs increased the expression levels of senescence-associated proteins (p53, p16 and p21) in a time-dependent manner (Figure 3B). Furthermore, the expression of cell cycle proteins, such as CyclinD1 and CyclinE1, was down-regulated. The expression of γ-H2AX, a marker molecule for DNA damage, was up-regulated (Figure 3C).

### MCCPs induced the ferroptosis in NRK-52E and HEK293 cells

**Figure 4:**
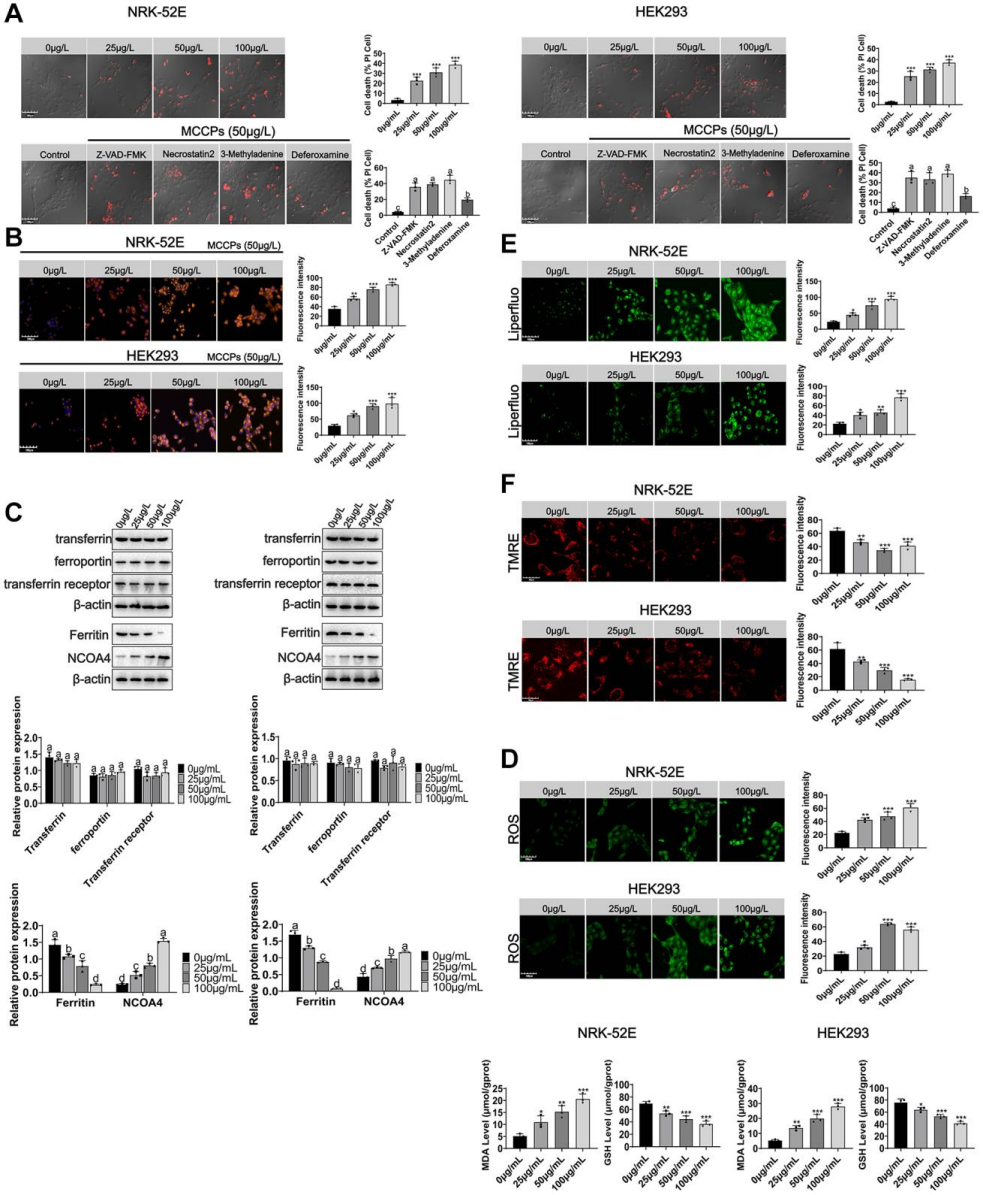
**MCCPs induced ferroptosis in renal cells.** (**A**) Analysis of the types of renal cell death induced by MCCPs. (**B**) MCCPs induced iron overload in renal cells. (**C**) Effects of MCCPs on transferrin, ferroportin, ferritin and NCOA4 expression. (**D**) Effects of MCCPs on oxidative stress. (**E**) Effects of MCCPs on lipid peroxidation. (**F**) Effects of MCCPs on mitochondrial membrane potential. *p* < 0.05 indicates significant differences.

Here, we investigated whether MCCPs could cause cell death. PI staining experiments showed that NRK-52E and HEK293 cells underwent death under MCCPs stimulation. To further determine the type of cell death, the inhibitors of apoptosis (Z-VAD-FMK), programmed necrosis (Necrostatin2) and autophagy (3-Methyladenine) were used to treat cells, respectively. It was found that these inhibitors did not significantly inhibit renal cell death induced by MCCPs. However, when an inhibitor of ferroptosis (Deferoxamine, DFO) was used, cell death was obviously reduced (Figure 4A). Therefore, we first evaluated the effect of MCCPs on iron ion levels within NRK-52E and HEK293 cells. We used a fluorescent probe for Fe^2+^ (FerroOrange), which showed that the levels of Fe^2+^ in NRK-52E and HEK293 cells were clearly elevated compared to control group (Figure 4B).

Next, we explored the reasons why MCCPs induced Fe^2+^ overload. The metabolic homeostasis of intracellular iron is regulated through transferrin and ferroportin. For this, we first explored the effect of MCCPs on transferrin and ferroportin expression, and found that the transferrin and ferroportin (FPN) expression was not changed after MCCPs treatment (Figure 4C). In addition, the transferrin receptor expression was not altered. The results of this part of the experiment indicate that the expression of ferritin, an intracellular iron storage protein, was significantly down-regulated. Furthermore, NCOA4-mediated autophagic degradation of ferritin, which is one of the most important factors for the induction of cellular Fe^2+^ overload, was found to be significantly up-regulated, which further contributed to the degradation of ferritin (Ferritin Heavy Chain, FTH), and up-regulation of the expression of NCOA4, which can promote FTH autophagy (Figure 4C).

ROS, GSH and MDA are also related markers of ferroptosis. Therefore, the effects of MCCPs on ROS, GSH and MDA levels within NRK-52E cell and HEK293 were investigated. The results showed that different concentrations of MCCPs induced an increase in ROS/MDA and a decrease in GSH levels. 50 μg/L MCCPs treated NRK-52E and HEK293 cells for 0 h-24 h. MCCPs could inhibit intracellular GSH production time-dependently when compared with the control group. Furthermore, MCCPs promoted the increase of MDA/ROS levels (Figure 4D). Additionally, we analyzed the lipid peroxidation of cells treated with MCCPs using a lipid peroxidation probe (Liperfluo), and experimental data showed that NRK-52E and HEK293 cells underwent lipid peroxidation under the treatment of MCCPs (Figure 4E).

Mitochondria are one of the major sources of cellular ROS production [15], so we evaluated the effect of MCCPs on mitochondrial membrane potential. The experimental results showed that different concentrations of MCCPs induced a decrease in mitochondrial membrane potential. Then, after we treated kidney cells with MCCPs at different time points, we found that the mitochondrial membrane potential was weakened, which is consistent with the characteristics of ferroptosis. Mitochondrial tracker staining showed disruption of mitochondrial integrity and increased fragmentation in MCCPs-treated cells, indicating severe mitochondrial damage (Figure 4F).

### Signaling pathways of ferroptosis induced by MCCPs

**Figure 5:**
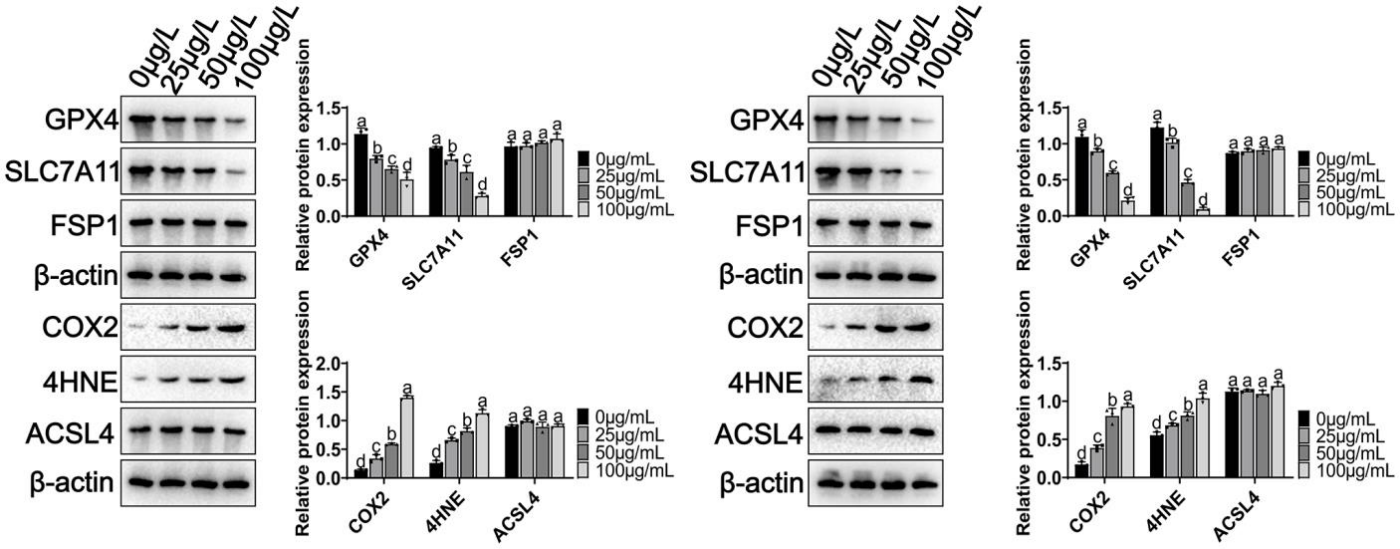
**Effect of MCCPs on ferroptosis-related signaling pathway in kidney cells.*** p* < 0.05 indicates significant difference.

We analyzed the signaling pathways involved in MCCPs-induced ferroptosis in kidney cells. First, System Xc--glutathione-GPX4 expression was analyzed, and experimental data showed a significant downregulation of GPX4, and SLC7A11 (xCT) expression (Figure 5). Recent studies have shown that FSP1-CoQ10 is also involved in the process of ferroptosis, so we also evaluated the expression of FSP1, and the results showed that FSP1 expression was not significantly changed in our experimental system. COX2 and 4HNE are important molecules reflecting the degree of intracellular lipid peroxidation damage, the experimental data illustrated that the levels of COX2 and 4HNE were obviously elevated. However, the expression of ACSL4 (which is involved in fatty acid synthesis) did not show significant changes (Figure 5).

### DFO alleviated MCCPs-induced ferroptosis in renal cells

**Figure 6:**
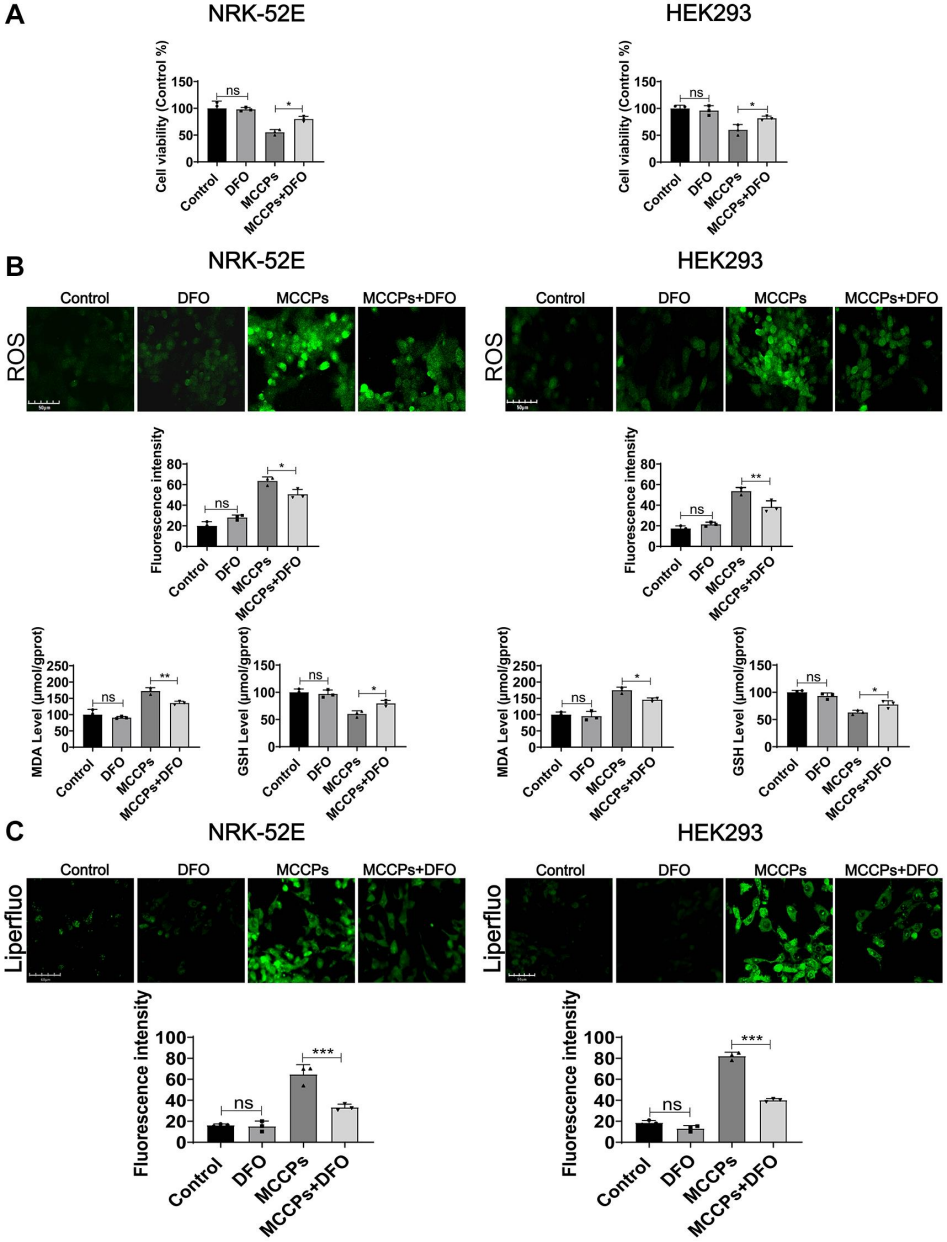
**Effect of DFO on cellular damage caused by MCCPs.** (**A**) Effect of DFO on cell viability. (**B**) Effect of DFO on ROS, MDA and GSH levels. (**C**) Effect of DFO on lipid peroxidation caused by MCCPs. *p* < 0.05 indicates significant difference.

To further determine the type of cell death induced by MCCPs belongs to ferroptosis, ferroptosis inhibitor (desferrioxamine, DFO) was used to treat the cells. DFO is an inhibitor of ferroptosis by neutralizing excess intracellular Fe^2+^ ions. The optimal concentration of action of DFO that does not affect cell viability was tested. NRK-52E and HEK293 cells were treated with DFO (0 μΜ-100 μΜ) for 24 h. The cell proliferation assays performed in pre-experiment showed that 10 μΜ of DFO did not show significant damaging effects on renal cells, so we chose 10 μΜ DFO as the optimal concentration to be used in the subsequent experiments. To investigate the effect of DFO on MCCPs-induced NRK-52E and HEK293 cell damage, we pre-treated kidney cells with 10 μΜ DFO for 5 h and then continued to treat cell with MCCPs for 24 h. The experimental data showed that the DFO pretreatment could significantly improve the cell viability compared with the MCCP-treated group (Figure 6A). In addition, oxidative stress analyses showed that ROS, MDA levels were significantly down-regulated, but GSH levels were increased under DFO treatment (Figure 6B). Lipid peroxidation probe (Liperfluo) levels were significantly decreased under DFO treatment (Figure 6C).

**Figure 7:**
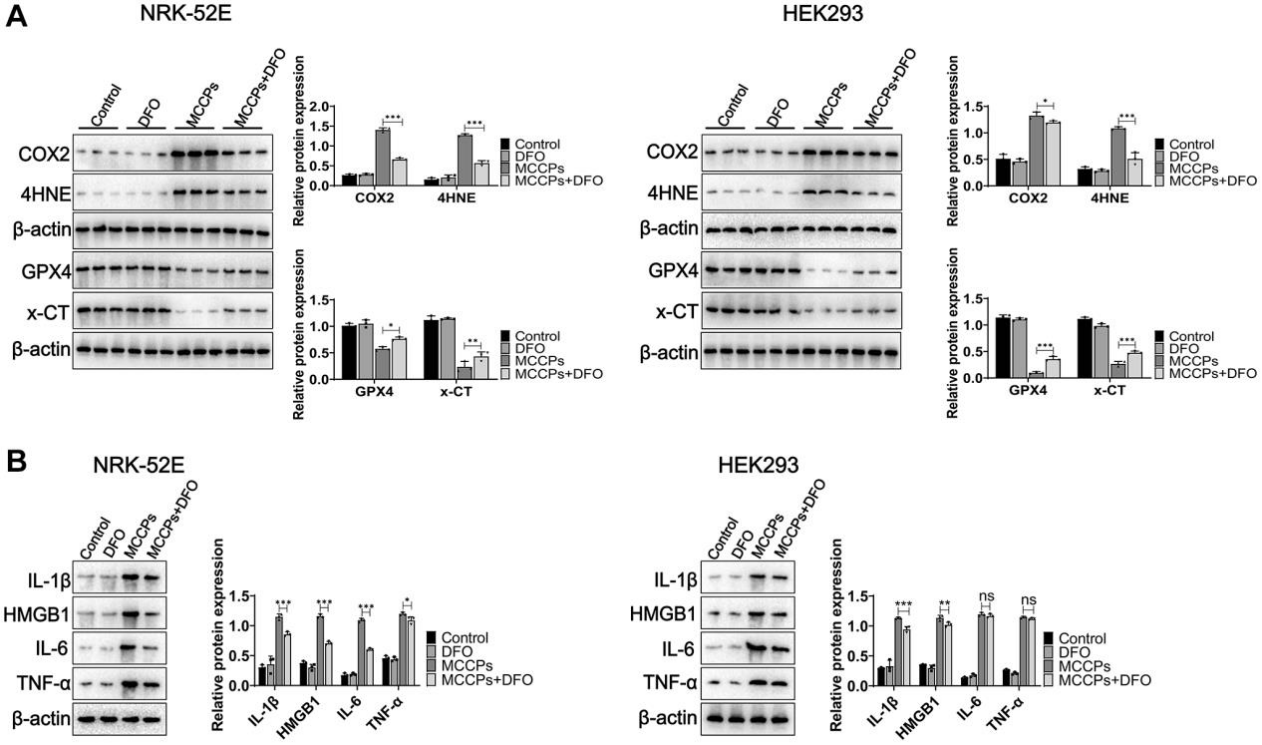
**DFO inhibited ferroptosis.** (**A**) Effect of DFO on the expression of MCCPs-induced ferroptosis-related proteins. (**B**) DFO alleviated the expression of inflammatory molecules. *p* < 0.05 indicates significant difference.

Further we investigated the effect of DFO on the expression of ferroptosis-related proteins. Western blot assays showed that x-CT and GPX4 expression was up-regulated under DFO treatment (compared with the MCCPs treatment group). However, COX2 and 4HNE expression was down-regulated (Figure 7A). Next, we analyzed the effect of MCCPs on the inflammation induced by MCCPs. The results showed that DFO alleviated the inflammatory response caused by MCCPs (Figure 7B).

### MCCPs-induced ferroptosis is central to cellular senescence

**Figure 8:**
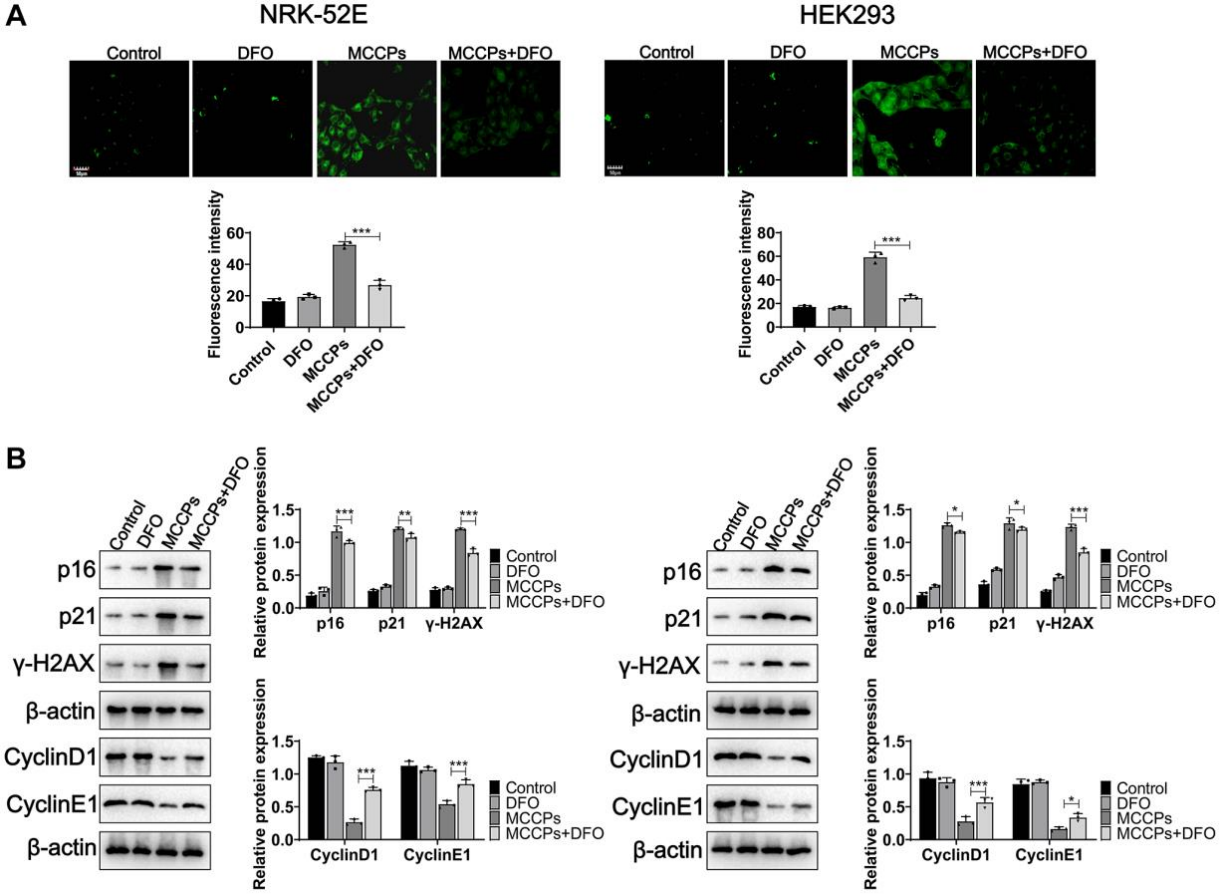
**DFO alleviated renal cell senescence caused by MCCPs.** (**A**) Sa-β-gal staining analysis of cell senescence. (**B**) Western blot was used to assess the effect of DFO on cellular senescence caused by MCCPs. *p* < 0.05 indicates significant difference.

To determine whether MCCPs-induced ferroptosis in renal cells is the basis of cell senescence, DFO was used to treat NRK-52E and HEK293 cells, the experimental results showed that they alleviated premature aging of renal cells by Sa-β-gal staining (SPiDER-βGal) (Figure 8A). Further experiments showed that DFO significantly reduced p21, p16, γ-H2AX expression, and increased CyclinD1 and CyclinE1 expression (Figure 8B).

### Assessment of the effect of MCCPs on the kidney tissue *in vivo*

**Figure 9:**
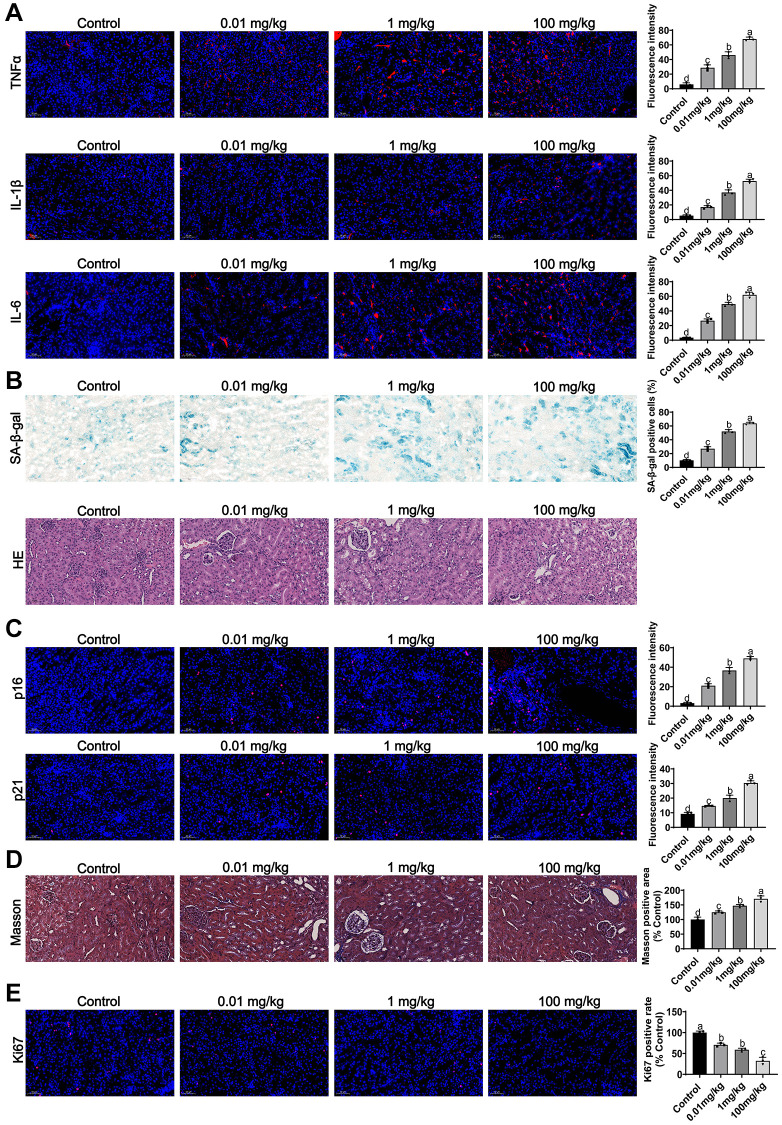
**Effects of MCCPs on renal inflammation and aging *in vivo*.** (**A**) Effect of MCCPs on renal inflammation. (**B**) Sa-β-gal and HE staining to analyze the effect of MCCPs on renal aging. (**C**) Effect of MCCPs on renal p21 and p16 expression. (**D**) Masson staining analysis of the effect of MCCPs on renal fibrosis. (**E**) The effect of MCCPs on Ki67 expression *in vivo*. *p* < 0.05 indicates a significant difference.

*In vivo*, we assessed the effect of MCCPs on renal inflammation and aging *in vivo*. The results of indirect immunofluorescence showed that MCCPs caused inflammatory responses (TNFα, IL-1β andIL-6) in the kidney (Figure 9A). Furthermore, we evaluated the effect of MCCPs on renal aging, and Sa-β-gal staining showed that MCCPs led to the renal aging. HE staining also indicated that renal tissue underwent damage (Figure 9B). Meanwhile, p21 and p16 staining also showed that MCCPs contributed to renal aging (Figure 9C). Furthermore, Masson staining showed that the level of kidney fibrosis was significantly increased compared to the control group (Figure 9D). Additionally, ki67 staining showed that the regenerative capacity of the kidney tissue was significantly reduced compared to the control group (Figure 9E). We also observed an elevation in iron ion levels in the MCCPs-treated group (Supplementary Figure 1).

## DISCUSSION

CPs are present in many media, including water, soil, air, and living organisms, etc. CPs can enter the animal body through the skin, the respiratory tract, and the gastrointestinal tract, and they exhibit rapid absorption and slow elimination [16]. A number of studies have confirmed the biotoxicity of SCCPs. Therefore, considering the toxic effects and environmental persistence of SCCPs, MCCPs are currently recommended to be used as a substitute for SCCPs. However, SCCPs and MCCPs have highly similar chemical structures, physicochemical properties and toxicological properties. There is not enough evidence to show that MCCPs are safe alternatives to SCCPs. In recent years, some studies have shown that the content of MCCPs is higher than that of SCCPs in some foods. Therefore, a systematic evaluation of the toxicokinetics and health risks of MCCPs is a future research priority in the field of toxicology. For this, the toxicological effects of MCCPs on the kidney were evaluated in the current work. We found that MCCPs caused cellular damage in the kidney, suggesting that MCCPs lead to the toxicity of kidney cells.

In this work, we found that MCCPs caused kidney cell death, and further studies showed that the type of cell death belonged to ferroptosis. Ferroptosis is a non-apoptotic form of programmed cell death discovered in recent years [17]. The concept of ferroptosis was first proposed in 2012 by Stockwell’s team, who discovered that erastin activates a particular mode of regulated cell death. They found that this mode of cell death is distinct from other forms of regulatory cell death (e.g., apoptosis, necrosis). It is a mode of cell death that results from iron-dependent lipid peroxidation and massive accumulation of reactive oxygen radicals (ROS). Therefore, Stockwell’s team first named this particular form of cell death as ferroptosis [18]. Subsequent series of studies have shown that ferroptosis is closely related to a variety of biological processes, including development, aging, immunity and cancer [19]. In the current work, we found that MCCPs led to a significant increase in intracellular free iron ion levels, suggesting that iron overload is one of the major molecular mechanisms by which MCCPs induce renal cell death. Further by evaluating ferroptosis-related marker molecules, we reconfirmed that the type of cell death induced by MCCPs belongs to ferroptosis.

Under normal physiological conditions, iron plays an extremely important role in the life activities of eukaryotic cells [20]. Fe^2+^ have several important biological functional roles in organisms. First, iron ions are an important component of hemoglobin and myoglobin, which transport oxygen to all parts of the body and maintain normal respiratory function [21]. Of course, an imbalance in the metabolism of iron ions in the cell will result in abnormal iron metabolism, which in turn will lead to a series of abnormal cellular reactions, such as ferroptosis [20]. The current work shows that MCCPs cause iron ions overload in kidney cells. Therefore, we inquire into the underlying mechanism by which MCCPs induce iron overload in kidney cells. In general, intracellular iron ion homeostasis can be regulated in several ways. Transferrin (responsible for transferring extracellular iron into the cell) and ferroportin (responsible for transferring intracellular iron to the outside of the cell). However, in our current work, we found that the expression of transferrin, ferroportin (FPN) and transferin receptor was not altered after MCCPs treatment. In addition, ferritin acts as an iron pool inside the cell. Ferritin can adsorb iron ions. We found a significant decrease in ferritin expression, which could provide an explanation for the iron overload under MCCPs treatment. However, recent studies have shown that the transferrin receptor (Slc39a14) mediates non-transferrin-bound iron into cell [22], which regulates the entry of iron ions into cells. But in the current work, we did not assess the expression of ZIP14. These findings suggests that the transport of iron ions is not unique.

We evaluated the effect of MCCPs on renal cellular senescence. We found that MCCPs led to renal cellular senescence by evaluating senescence markers, such as Sa-β-gal and p16/p21. Further studies showed that ferroptosis is a potential molecular pathway for MCCPs to induce inflammation and senescence. Previous studies have shown that ferroptosis is closely associated with aging, such as Alzheimer’s disease (AD), Parkinson’s disease (PD), cardiovascular diseases (CVDs), diabetes, and chronic inflammation [23]. Thus, inhibition of ferroptosis may alleviate aging-related diseases. Furthermore, in the current work, we explored whether DFO could inhibit ferroptosis caused by MCCPs, and the results showed that DFO could alleviate renal aging and ferroptosis caused by MCCPs.

The current toxicological studies imply that these MCCPs may not be the best safe alternative to SCCPs. However, the currently available toxicological data are still limited, and a more systematic toxicity assessment of MCCPs is needed in future work. In conclusion, this work provides a basic theoretical basis for the potential ecological risk assessment of MCCPs, and also provides basic experimental data for the rational and standardized use of MCCPs.

## CONCLUSION

Here, we investigated the toxicological effects of MCCPs on kidney cells, revealing that MCCPs induced ferroptosis and senescence in these cells. This study offers a fundamental theoretical framework for the potential ecological risk assessment of MCCPs and provides essential experimental data for their rational and standardized utilization.

## Supplementary Materials

Supplementary Figure 1
