# Supplementary figures and images for: Medium-chain chlorinated paraffins (MCCPs) induce renal cell aging and ferroptosis
Source: Aging (Albany NY). 2024 Apr 19;16(8):7277–92. doi: 10.18632/aging.205756 (PMC11087104; doi:10.18632/aging.205756)

## SUPPLEMENTARY FIGURE

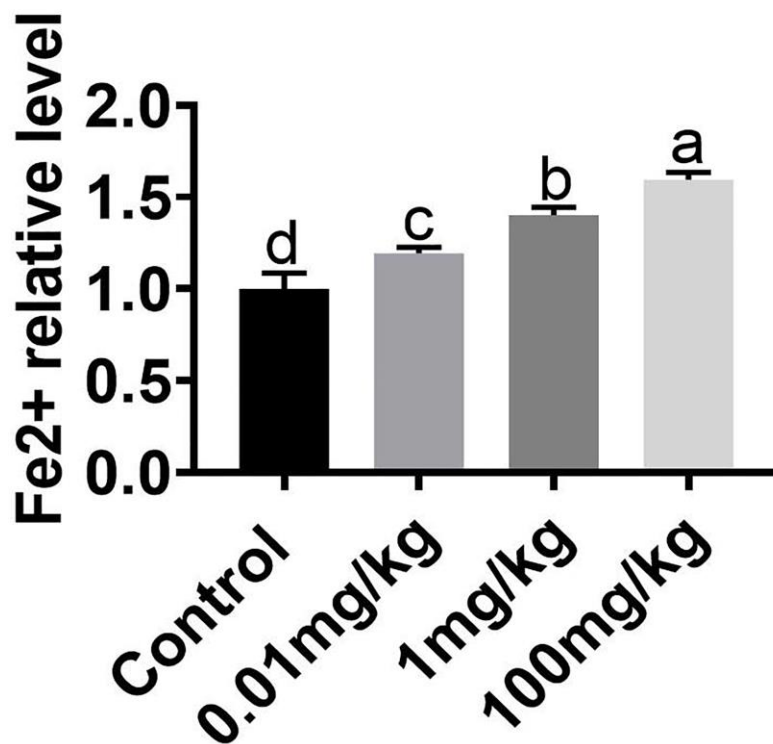

Supplementary Figure 1. Detection of the level of iron ions.

Supplement: Supplementary Figure 1 [file aging-16-205756-s001.pdf]
